# Evaluation of Two Spectro-Temporal Ripple Tests and Their Relation to the Matrix Speech-in-Noise Sentence Test in Cochlear Implant Recipients

**DOI:** 10.1097/AUD.0000000000001365

**Published:** 2023-04-13

**Authors:** N.R.A. van Groesen, J.J. Briaire, J.H.M. Frijns

**Affiliations:** 1 Otorhinolaryngology and Head and Neck Surgery, Leiden University Medical Center, the Netherlands; 2 Leiden Institute for Brain and Cognition, Leiden, the Netherlands

**Keywords:** Cochlear Implant, Psychophysics, Speech perception, Spectral ripple, Spectro-temporal ripple

## Abstract

**Objectives:**

Spectro-temporal ripple tests are commonly used in cochlear implant (CI) research as language-independent indicators of speech recognition (in noise) or as stand-alone tests. Test-retest reliability of these tests has been scarcely documented. We evaluated the test-retest reliability of spectral-temporally modulated ripple test (SMRT) and spectro-temporal ripple for investigating processor effectiveness (STRIPES) and correlated their findings to the Dutch/Flemish Matrix speech-in-noise sentence test (MST) in CI recipients. This is the first time spectro-temporal ripple tests are correlated to an MST.

**Design:**

Take-home data from 15 participants over 2 test days were analyzed. Participants were fitted with their clinical speech encoding strategy (Advanced Bionics HiRes Optima) or a 14-channel non-steered monopolar strategy. Test-retest reliability was calculated through intraclass correlation coefficients and visualized through Bland Altman plots. Association of the spectro-temporal ripple tests with the MST was evaluated through linear regression analysis.

**Results:**

The SMRT and STRIPES possessed a similarly rated “good” test-retest reliability (SMRT: ICC = 0.81, confidence interval = 0.67 to 0.92; STRIPES: ICC = 0.87, confidence interval = 0.76 to 0.95) and an identical linear relationship to speech recognition in noise (SMRT: *R*^2^ = 0.28, *p* = 0.04; STRIPES: *R*^2^ = 0.28, *p* = 0.04). Both tests revealed a stable variability between session 1 and 2 outcome scores on Bland Altman plots.

**Conclusion:**

On the basis of our data, both spectro-temporal ripple tests possess similar test-retest reliability and a similar association with the MST. The SMRT and STRIPES can therefore both be used equally well as a quick indicator of across-listener differences in speech recognition in noise in CI recipients.

## INTRODUCTION

Cochlear implants (CIs) have a good track record of restoring speech recognition in quiet for post-lingually deafened participants, although high variability in performance still persists among individuals (Firszt et al. 2004). As one of its causes, degraded spectral resolution and reduced spectral selectivity of CI recipients have been identified as causes of poor speech recognition (Henry & Turner 2003; Donaldson et al. 2005; Henry et al. 2005; Won et al. 2007; Azadpour & McKay 2012). To evaluate spectral resolution, several types of spectral and spectro-temporal ripple tests have been developed and are now commonly found in CI research.

Spectral and spectro-temporal ripple tests have been correlated to speech recognition in quiet and speech recognition in noise and can serve as a quick indicator of CI performance (Won et al. 2007; Drennan et al. 2014; Jeon et al. 2015; Holden et al. 2016; Lawler et al. 2017; Goehring et al. 2019). However, not all researchers have found this correlation of spectral ripple tests to speech recognition to hold true (Anderson et al. 2011; Azadpour & McKay 2012).

An added benefit of non-speech tests is that the outcomes are not susceptible to language barriers and therefore allow for comparisons of data across language borders.

In general, a spectral ripple is defined as a broadband stimulus consisting of multiple sinusoids covering a certain frequency range, modulated on a logarithmic or linear frequency scale (Supin et al. 1998; Won et al. 2007). The stimulus has peak-to-valley amplitude fluctuations along the frequency axis. The test outcome scores are presented as ripple density (RD) when implemented on a linear frequency axis and ripples per octave (RPO) when a logarithmic scale is used (Narne et al. 2016). If the stimulus shifts across multiple frequencies over time, it is considered to be a spectro-temporal test. In a spectro-temporal test, participants must make comparisons between stimuli in the spectral as well as the temporal domain, making it less likely for them to solely focus on spurious cues present in either of the two domains itself.

In this study, we compared the spectro-temporal ripple for investigating processor effectiveness (STRIPES) and the spectral-temporally modulated ripple test (SMRT), two spectro-temporal tests based on different foundations. The STRIPES (Archer-Boyd et al. 2018) is a glide direction discrimination task in which an upsweeping target stimulus is compared with two downward sweeping reference stimuli of the same RD. The goal is to find the highest RD at which a participant can correctly discriminate upward from downward gliding ripples.

The SMRT by Aronoff and Landsberger (2013) is an RD discrimination task. In this test, the target and reference stimuli consist of ripples with an identical glide direction but contain a different density between the successive ripples. Participants aim to find the point at which they can successfully discriminate the smallest distinguishable density between target and reference stimuli, where the reference has a very high spectral density.

Other spectral ripple test variants include ripple phase discrimination tests and spectral ripple detection tests. In ripple phase discrimination tests, the target and reference stimuli have the same RD, but a phase shift creates a distinguishable difference. One such test is the spectral ripple test from Won et al. (2007).

Spectral ripple detection tasks require participants to differentiate a ripple-modulated stimulus from an unmodulated stimulus. By varying the spectral modulation depth, the outcome is the point where the modulated carrier can no longer be discriminated from the unmodulated carrier. Examples can be found in the psychophysical tests of Litvak et al. (2007) and Azadpour and McKay (2012).

Not all researchers agree that spectral or spectro-temporal ripple discrimination tests accurately measure spatial resolution as intended. Spurious cues, such as loudness differences, spectral centroid shifts and changes to spectral edges can drive performance on spectral ripple discrimination tasks (Supin et al. 1998). In addition, O’Brien and Winn (2017, 2022) simulated that a CI processor probably cannot correctly transmit spectral densities above a certain critical limit. By simulating the output of a 22-channel Cochlear Nucleus processor, they found that above the approximate critical point of 2.56 RPO, processor output becomes distorted. As a result, the stimuli become similar to the modulation spectrum of vowels, leading to new cues and an entirely different perceptual criterion to be evaluated. In line with O’Brien and Winn (2017), Azadpour and McKay (2012) claimed that CI recipients cannot rely on fine spectral information for speech understanding and that spectral cues, contextual cues, or temporal information probably drive across-listener differences in speech recognition.

To account for frequency-specific onset and offset cues in the chosen spectrum, the SMRT’s onset phase is randomized for all stimuli. In addition, the modulation phase drifts with time to minimize loudness level differences per specific frequency or electrode. However, for low target densities (<2 RPO), loudness differences on one channel between a low-ripple target and a high-ripple reference stimulus can become large enough to allow for discrimination (Archer-Boyd et al. 2018). Using a spectral RD discrimination and spectro-temporal ripple test based heavily on the SMRT, Narne et al. (2016) found higher performance on their spectro-temporal ripple test than on the spectral ripple test in normal hearing listeners. When adding bookend noises to the outsets of both, the scores were similar. This suggests that a useful cue for discriminating between target and reference stimuli was present in the outsets of the spectro-temporal stimulus, even with randomization of the onset phase and drifting of the modulation phase.

In an attempt to overcome these spurious cues present in spectral ripple testing, Archer-Boyd et al. (2018) created the STRIPES. This test contains equal ripple densities for target and reference stimuli, a roved onset frequency, and bookend noise added on the outsets of each stimulus. With identical ripple densities for each electrode, the loudness dips and spectral centroids between the target and reference are identical. Roving the onset phase and adding bookend noise reduces the possibility of onset and offset cues.

In a spectro-temporal test based on the STRIPES, Narne et al. (2018, 2019) found that the test was not affected by cues related to changes at the spectral edges and, therefore, provided a reliable estimate of frequency resolution as long as the repetition rate did not exceed 8 Hz. With a repetition rate of 5 Hz the STRIPES are expected to be free from cues at the spectral edges.

In the present study, the SMRT and STRIPES will be correlated to the Matrix speech-in-noise sentence test (MST). The MST is often used in our clinic to evaluate speech recognition in noise. The MST was first developed by Hagerman (1982) and consists of a matrix of names, verbs, numerals, adjectives, and nouns. Syntactically, the structure is identical for all sentences, but semantically the content is unpredictable (Hagerman 1982; Houben et al. 2014). The most acknowledged disadvantage of the MST is that the fixed design does not mimic true everyday situations, losing some logical semantic meaning in its sentences. However, the MST does resemble real-life situations better than individual word recognition testing and makes repeatable testing more comparable to pure sentence recognition testing.

The SMRT and STRIPES have both been correlated to word and sentence recognition before, both in quiet and in noise (Holden et al. 2016; Lawler et al. 2017; Landsberger et al. 2018; Goehring et al. 2019), but to the best of our knowledge, no correlation to an MST has yet been described in literature. In fact, the MST has never been correlated to any spectro-temporal or spectral test. In the present study, we evaluated the test-retest reliabilities of the SMRT and STRIPES and assessed their correlation with the Dutch/Flemish MST using a conventional steered clinical strategy (Advanced Bionics HiRes Optima) and monopolar stimulation (MP). On the basis of literature (O’Brien & Winn 2017; Winn & O’Brien 2022) casting doubt on the ability of the SMRT to correctly present a stable measure of spectral resolution at higher ripple densities, we expect the STRIPES to present a better test-retest reliability and a more stable association with the MST compared with the test-retest reliability of the SMRT and its association with the MST.

## MATERIALS AND METHODS

### Study Design

Two test sessions spaced 6 weeks apart were conducted in a single-blinded design. In each session, participants completed three runs of the SMRT, the STRIPES, and the MST in randomized order. The 14-channel MP data were extracted from a recently conducted take-home trial consisting of the same design (Van Groesen et al. 2023).

Six participants completed the trial with their clinical Advanced Bionics Hires Optima strategy (Advanced Bionics 2012). With the addition of 9 participants with MP data, total data of 15 participants was available for analysis.

The study protocol was approved by the Committee for Medical Ethics of Leiden University Medical Centre (P02.106).

### Participants

Participant characteristics are presented in Table 1. All participants were implanted with Advanced Bionics HiRes90K HiFocus 1J or HiRes90K HiFocus Mid-Scala (MS) electrode arrays (5 1J, 10 MS). The mean age was 66.8 (SD 5.2) years in the clinical group and 61.4 (SD 11.0) years in the MP group (t = 1.1, df = 13, *p* = 0.3). Mean most recent word recognition scores (clinical group: 75.0% SD 9.1; MP: 76.4% SD 8.5) and mean consonant-vowel-consonant scores (clinical group: 87.8% SD 4.4; MP: 90.8% SD 4.9) were not significantly different between the clinical group and the MP group (respectively, t = −0.3, df = 13, *p* = 0.8 and t = 1.3, df = 13, *p* = 0.2). Mean duration of deafness was 17.0 years (SD 14.2) in the clinical group and 22.5 years (SD 12.9) in the MP group (t = 0.8, df = 13, *p* = 0.6). Only phase width was significantly different between the clinical group (mean 29.4 μs, SD 8.0) and the MP data (mean 50.9 μs, SD 13.7) (t = −3.8, df = 13, *p* = 0.002). This was inherently due to the different configurations of the strategies.

**TABLE 1.** Participant characteristics

Participant	Allocation	Electrode Array	Gender	Age (years)	CI Side	Etiology	Implant Use (years)	Deafness (years)	Word (%WS)	CVC (%PH)	Rate (pps)	PhW (µs)
ID01	HiRes Optima	MS	Female	69	AD	Progressive	2	30	75	89	3093	21.6
ID10	HiRes Optima	MS	Male	74	AS	Progressive	2	12	80	93	1768	37.7
ID17	HiRes Optima	MS	Female	67	AD	Sudden Deafness	3	6	78	88	2007	33.2
ID22	HiRes Optima	MS	Female	58	AS	Genetic	2	2	57	80	3093	21.6
ID25	HiRes Optima	MS	Female	70	AS	Genetic	3	11	78	87	1727	38.6
ID28	HiRes Optima	1J	Male	63	AS	Progressive	12	43	82	90	2750	24.2
ID04	MP	1J	Female	66	AS	Genetic	9	15	77	91	1459	52.1
ID08	MP	1J	Female	46	ADS (AD)	Meningitis	8	8	86	95	1461	66.4
ID09	MP	MS	Male	45	AD	Progressive	2	35	76	95	1206	43.1
ID13	MP	MS	Male	72	AS	Progressive	3	23	76	90	980	35.0
ID18	MP	MS	Male	70	AS	Progressive	6	44	80	92	1736	62.0
ID19	MP	1J	Female	72	AS	Progressive	10	15	75	90	1682	64.7
ID23	MP	1J	Female	71	AS	Cholesteatoma	3	35	61	80	1612	62.0
ID24	MP	MS	Female	62	AS	Progressive	1	3	68	88	980	35.0
ID27	MP	MS	Male	49	AD	Progressive	2	25	89	96	980	35.0

HiRes Optima, Advanced Bionics HiRes Optima; MP, monopolar stimulation; MS, HiRes90K HiFocus Mid-Scala; 1J, HiRes90K HiFocus 1J; AS, left ear; AD, right ear; ADS (AD), both ears, AD, tested ear; CVC, consonant-vowel-consonant phoneme score; Deafness, duration of deafness in years; Word; word score; %WS, percentage of words correct on standardized word recognition test at 65 dB HL; %PH, percentage of phonemes correct on standardized monosyllabic word test at 65 dB HL; Rate, pulse rate; PhW, phase width.

published online ahead of print April 13, 2023.

### Speech Encoding Strategies

HiRes Optima is a dual-electrode, sequentially current steered strategy, and the present clinical standard for Advanced Bionics speech processors. It is a modified version of the HiResF120 strategy. By limiting current steering to a more central position of the two electrodes (*α* between 0.25 and 0.75), it efficiently saves energy without loss of speech recognition (Advanced Bionics 2012). The *α* denotes the fraction of current directed to the apical electrode, and 1–*α* is concurrently sent to the apical electrode. The clinical strategy of each participant’s most recent clinical visit was copied and fitted on a Harmony research processor through SoundWave software (Advanced Bionics, Valencia, CA). Noise cancellation features, if active, were turned off throughout the study.

The MP group received a 14-channel MP strategy through a conventional clinical fitting of threshold levels and most comfortable levels. Afterward, these were evaluated and minimally adjusted based on loudness perception, sound quality, and background noise.

### Psychophysical Tests

All tests were conducted at 65 dB SPL in the free field in a double-walled, sound-attenuating booth. The participant faced a single loudspeaker at 0° azimuth at a distance of 1 m. Psychophysical tests were calibrated to 65 dB SPL using a handheld sound level meter (Voltcraft SL-200, Hirschau, Germany). In the MST, speech and noise were produced by the same loudspeaker and the noise varied adaptively. For each psychophysical test, participants completed three runs per session and a total of six runs over two sessions.

### SMRT

The SMRT (Aronoff & Landsberger 2013) determines the threshold at which a participant can discriminate a 500 ms target stimulus from two reference stimuli of 20 RPO. The target stimulus starts at 0.5 RPO and RD increases in 0.2 RPO steps. The stimuli span 100 to 6400 Hz. The onset phase for each stimulus is randomized and the modulation phase drifts with time to minimize differences in loudness levels for each specific frequency or electrode. The SMRT is a three-interval, three-alternative forced-choice task using a one-up/one-down adaptive procedure and takes 10 reversals to complete. Thresholds are calculated based on the average of the last six reversals. The test was controlled by custom MATLAB software (MathWorks, Natick, MA). The outcome is presented in RPO.

### STRIPES

The STRIPES test (Archer-Boyd et al. 2018) determines the threshold at which participants can identify a 1-sec upward-sweeping sinusoidal stimulus in reference to two 1-sec downward-sweeping sinusoidal stimuli of the same RD at a rate of 5 octaves per second. This means that an RD of 1.0 corresponds to an RPO of 0.2. The target stimulus starts at an RD of 1.1 (number of sweeps per 1-sec interval) and increases by two steps of 0.5 for the first four reversals and 0.2 for the last eight reversals. The stimuli span 250 to 8000 Hz. The starting frequency of each stimulus is roved, and each stimulus is bookended by 250 ms of noise, with a 125-ms crossfade to minimize differences in loudness levels on the outsets of the stimuli. The STRIPES is a three-interval, two-alternative forced-choice task using a two-up/one-down adaptive procedure and takes twelve reversals to complete. Threshold density is calculated based on the average of the last four reversals. This test was also controlled by custom MATLAB software (MathWorks, Natick, MA). The outcome is presented in RD.

### MST

The MST is a matrix-oriented adaptive speech test (Hagerman 1982; Wagener et al. 1999) that determines the speech reception threshold (SRT) in speech-weighted noise. The SRT is the average point in dB signal to noise ratio (SNR) at which a test-taker recognizes approximately 50% of a 5-word sentence (i.e., the average SNR between 2 of 5 and 3 of 5 correct). The Flemish/Dutch test, spoken by a single female talker, comprises 50 unique words consisting of 10 names, 10 verbs, 10 numerals, 10 colors, and 10 objects. Thirteen balanced lists of 20 sentences containing 5 words each were used in open-set testing. The speech level was adaptively varied using a staircase procedure. A reduction in step size was dependent on the number of reversals and the score obtained in the previous trial (Francart et al. 2008). Step size varied from 2 at the start to 0.1 or 0.2 dB at the end of the trial. Participants were instructed to repeat the five words and to guess if they were unsure. In each test session, three lists were randomly selected, and the lists were never repeated in the same test session. In each session, 10 randomly selected practice sentences without noise were completed to allow for acclimatization to the test. Speech-to-noise ratio started at −4 dB SPL. The task was carried out using APEX 3 software (Leuven, Belgium) installed on a personal computer (Francart et al. 2008). The outcome is presented as SRTs in dB SNR.

### Missing Data

Participants ID01 and ID22 completed fewer formal runs of the STRIPES (two per session instead of three). ID01 performed the test at ceiling level from the first run onwards. ID22 performed fewer runs because of time constraints.

### Statistical Analysis

Test-retest reliability was estimated through intraclass correlation coefficients (ICCs) based on six runs (three per session) and their 95% confidence intervals based on absolute agreement and two-way mixed-effects models (Fisher 1925; Bartko 1966; Koo & Li 2016) (Equation 1).

$$\begin{array}{l} ICC=\;\frac{MSBP-MSE}{MSBP+(k-1)MSE+\left(\frac{k(MSBM-MSE)}{n}\right)} \end{array}$$ (1)

where MSBP is the mean square between participants’ outcome scores, MSE is the mean square for error, *k* is the number of measurements, MSBM is the mean square between measurements, and *n* is the number of participants. The ICC is a commonly used index reflecting both the degree of correlation and the agreement between measurements. ICC values <0.5 are indicative of poor reliability, values between 0.5 and 0.75 indicate moderate reliability, values between 0.75 and 0.9 indicate good reliability, and values >0.90 indicate excellent reliability (Portney & Watkins 2009).

The agreement between session 1 and session 2 data was visualized through a Bland Altman plot (BA plot) (Bland & Altman 1986, 1995; Giavarina 2015). A BA plot visualizes the offset between session 1 and session 2 scores along with the weighted mean score of both sessions.

The association of the STRIPES and SMRT with the MST was achieved using linear regression analysis. Correlations are presented as *R*^2^ values and *p* < 0.05 was considered significant.

Statistical analyses were performed using SPSS Statistics for Windows, Version 26.0 (IBM, Armonk, NY).

## RESULTS

### Test-Retest Reliability

On the SMRT, the mean score was 3.7 RPO (SD 2.4 RPO). The clinical group mean score was 4.4 RPO (SD 2.6 RPO) and the MP group mean score was 3.3 RPO (SD 2.2 RPO). The overall mean within-subject variability range was 2.5 RPO (SD 1.6 RPO; minimum range 1.3 RPO, maximum range 6.5 RPO). The overall ICC was.81 (*p* < 0.001; 95% confidence interval = 0.67 to 0.92). When analyzing the two subsets the clinical group revealed an ICC of 0.82 (*p* < 0.001; 95% confidence interval = 0.58 to 0.97) and MP revealed an ICC of 0.81 (*p* < 0.001; 95% confidence interval = 0.62 to 0.94). In all three analyses, this translates into good reliability with a confidence interval ranging from moderate to excellent.

In the STRIPES analysis, the mean score was 4.8 RD (SD 2.3 RD) with a clinical group mean score of 6.5 RD (SD 2.4 RD) and an MP group mean score of 3.7 (SD 1.6 RD) in the MP group. The mean range of within-subject variability on the STRIPES was 2.0 RD (SD 1.1 RD) with a minimum range of 0 RD and a maximum range of 3.5 RD. The test-retest reliability was calculated to an ICC of 0.87 with a 95% confidence interval of 0.76 to 0.95 (*p* < 0.001).

The clinical group revealed an ICC of 0.90 (*p* < 0.001; 95% confidence interval = 0.75 to 0.98), and the MP group revealed an ICC of 0.72 (*p* < 0.001; 95% confidence interval = 0.47 to 0.91). The overall ICC translates into good reliability with a confidence interval of moderate to excellent, similar to the SMRT. However, the MP group scored moderate reliability with a confidence interval ranging from poor to excellent.

The overall MST mean score is 1.9 dB SNR (SD 3.0 dB), with 1.6 dB SNR (SD 2.5 dB) in the clinical group and 2.2 dB SNR (SD 3.4 dB) in the MP group. The mean within-subject variability range on the MST was 4.3 dB, with a minimum range of 2.3 dB and a maximum range of 8.1 dB. The overall ICC was 0.72, with a confidence interval ranging from 0.53 to 0.87 (*p* < 0.001). The clinical group ICC was 0.70 (*p* < 0.001; 95% confidence interval = 0.39 to 0.94) and the MP group’s ICC revealed as 0.74 (*p* < 0.001; 95% confidence interval = 0.49 to 0.92). These outcomes translate into moderate reliability, ranging from moderate to good in the overall analysis but ranging from poor to excellent in the subset analyses.

### Bland Altman Plot

**Fig. 1.**
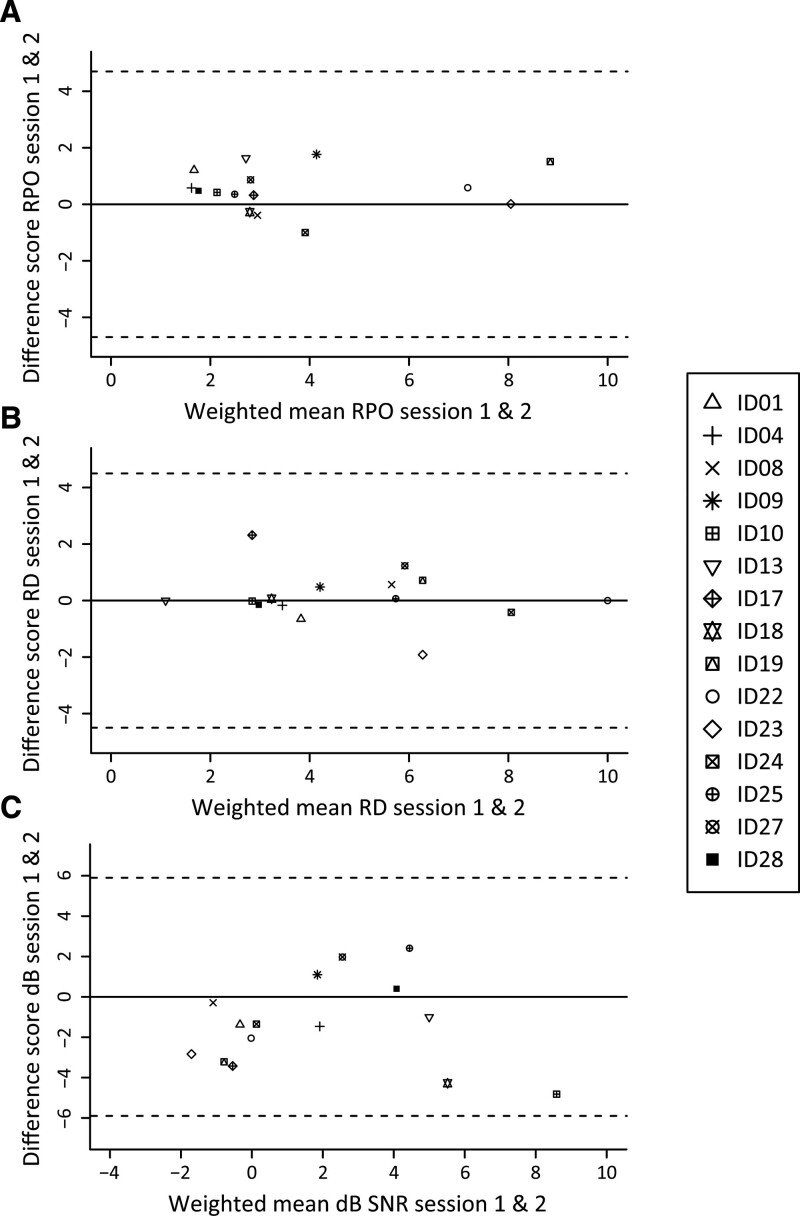
Bland Altman plots with session 1 and session 2 outcome data of (A) SMRT (B) STRIPES and (C) MST. MST indicates matrix speech-in-noise sentence test; SMRT, spectral-temporally modulated ripple test; STRIPES, Spectro-temporal ripple for investigating processor effectiveness.

On the SMRT BA plot (Fig. 1A), the nonsignificant linear relation between offset data and the weighted mean data reveals that no skewing is present between session 1 and 2 outcomes (F(1,13)= 1.5, *p* = 0.7; *R*^2^ = 0.009). Visually, no funneling of data is present. Similar variability is present along the abscissa in both the lower as well as the higher scores. All data fall within the 95% confidence interval.

The STRIPES BA plot (Fig. 1B) also reveals a stable relation between session 1 and session 2 data. The regression analysis revealed no deviation from the abscissa (F(1,13) = 5.6, *p* = 0.55; *R*^2^ = 0.03). All data fall within the 95% confidence interval.

The MST BA plot (Fig. 1C) reveals that participants who performed either poorly (high mean SRT) or better than average (low mean SRT) in session 1 improved in the second session (i.e. negative offset scores). A quadratic regression analysis revealed a significant interaction between the offset and weighted mean of session 1 and session 2 MST data (F(1,12) = 5.5, *p* = 0.02; *R*^2^ = 0.47). Hereby revealing that the MST data contains significant variability differences along the abscissa en therefore does not have a stable relation between sessions 1 and 2. All data fall within the 95% confidence interval.

### Association Analyses

**Fig. 2.**
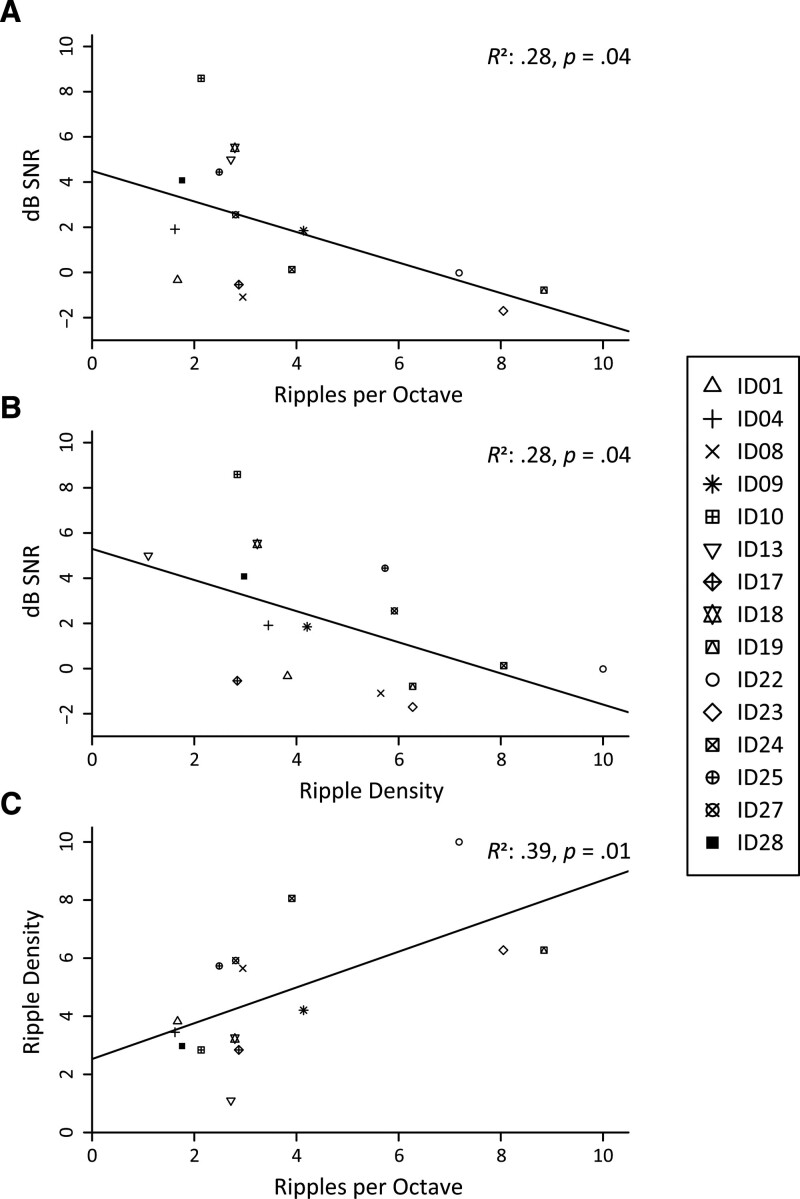
Plots of linear regression analyses correlating (A) SMRT and MST (B) STRIPES and MST. MST indicates matrix speech-in-noise sentence test; SMRT, spectral-temporally modulated ripple test; STRIPES, Spectro-temporal ripple for investigating processor effectiveness.


As visualized in Figure 2A, a significant linear regression equation was found for mean SMRT outcomes in relation to the MST (F(1,13) = 6.9, *p* = 0.04; *R*^2^ = 0.28). The predicted SRT in dB SNR is presented in Equation 2:

$$\begin{array}{l} SRT=4.5-0.68*RPO \end{array}$$ (2)

For every gain of 1 RPO on the SMRT, the predicted SRT decreased (i.e. improved) by 0.68 dB SNR.

On the basis of the simulations of O’Brien and Winn (2017), we intended to analyze data <2.56 RPO. However, only four participants had mean outcome scores below this threshold, so no meaningful analysis could be completed.

When correlating the mean STRIPES scores to mean MST scores (Fig. 2B), we found a significant regression equation (F(1,13) = 6.9, *p* = 0.04; *R*^2^ = 0.28), presented in Equation 3:


$$\begin{array}{l} SRT=5.3-0.69*RD \end{array}$$ (3)

For every improvement of 1 RD on the STRIPES, SRT is expected to decrease (i.e.: improve) by 0.69 dB SNR.

## DISCUSSION

This study investigated two spectro-temporal ripple tests and their relationship to speech recognition in noise (the Dutch/Flemish MST). The test-retest reliability was rated “good” for both tests with confidence intervals ranging from moderate to excellent. Yet, the within-subject variability of outcomes was considerable for some participants. We found no other ICCs of the SMRT and STRIPES in literature to which we can compare these findings and proper test-retest reliability analyses are scarce. Through a mathematical variant of the BA plot, Goehring et al (2019) found variability of both the SMRT and STRIPES to be stable. Archer-Boyd et al. (2020) visualized the STRIPES data of that study in a BA plot and confirmed these findings. Archer-Boyd et al. (2018) found that pairs of thresholds in normal hearing participants differ by <1.0 RD on the STRIPES; most by <0.5 RD based on two-tailed *t*-tests. In CI recipients, the threshold difference was also reported to be <0.5 RD for most.

Goehring et al. (2019) determined test-retest reliability of the STRIPES and SMRT by correlating the mean scores of three runs of each psychophysical test on two separate sessions. Both the STRIPES and SMRT were considered to have strong test-retest reliability based on these correlations (*R* = 0.91 and *R* = 0.87, respectively). Our study found a similar *R* for correlation of the STRIPES to speech recognition in noise as Goehring et al. (2019) (this study’s *R* = 0.53 vs. 0.59) but a remarkably higher *R* for the SMRT in relation to speech recognition in noise (this study’s *R* = 0.53 vs. 0.33 in literature). In the cited study, eight post- or peri-lingually deafened participants were tested with Bamford-Kowal-Bench sentences mixed with time-reversed speech sentences drawn from the Harvard sentences (IEEE 1969; Bench et al 1979). Although there were differences in the study set-up between their study and this one (e.g., choice of speech-in-noise recognition test, gender of speaker, direct auxiliary input vs. free field testing). Archer-Boyd et al. (2020) later compared the effect with direct auxiliary input of stimuli into the Advanced Bionics Harmony Research processor to a loudspeaker setting in 10 subjects and found the variability in STRIPES outcomes between the two settings stable along the abscissa of a BA plot as well.

The only other reported test-retest reliability of spectro-temporal ripples was performed by Drennan et al. (2014) for the ripple discrimination test of Won et al. (2007). Through regression analysis, they found a strong correlation (*R* = 0.90) and therefore concluded excellent agreement. However, linear regression analyses and *t*-tests measure association and significance, respectively, not agreement, and are unsuitable measures for test-retest analyses. It is known that *t*-tests do not measure within-subject changes, only group changes. Regression analyses only measure the strength of a linear relationship between two variables. Even variables with poor agreement can have relatively strong correlation coefficients (Altman & Bland 1983; Bland & Altman 1986; Giavarina 2015). The ICC can be used in studies of test-retest reliability, as it is based on analysis of variance and the estimation of various components of variance (Bartko 1966). ICCs account for within-subject changes, as well as average changes in group performance over time (Vaz et al. 2013; Koo & Li 2016; Vetter & Schober 2018). There are many different versions of the ICC, each tailored to specific research designs (Shrout & Fleiss 1979). We would advocate the use of correct ICCs in future validation studies of new psychophysical tests to allow for quick and easy presentation of a test’s test-retest reliability.

Despite the inherent variability, both the SMRT and STRIPES reveal a moderate linear relationship with the MST when tested in separate correlation analyses. In addition, the slopes of the regression lines were virtually identical. This could mean that the differences in spurious cues between the SMRT and STRIPES play little to no role in estimating across-listener differences in speech recognition in noise.

Speech-in-noise recognition is test known to be susceptible to within-subject variability. This can be due to learning effects over time or other unspecified cues. The test-retest variability of the MST ranges from 0.4 to 0.7 dB for various European languages in normal hearing participants (Kollmeier et al. 2015; Willberg et al. 2020). These scores are based on the used root mean square value of the differences between two SRTs divided by √2 (Plomp & Mimpen 1979; Willberg et al. 2020). The SRT variability in this study would be 1.1 dB when comparing the mean outcomes of session 1 with session 2. This shows that on the MST, CI recipient variability is worse than the average variability of normal hearing users as found in literature.

Interesting to note is the fact that the SMRT was completed in 3 to 7 min, whereas the STRIPES test required 7 to 25 min. Whether this difference had any effect on the reliability of the outcomes is unknown. For two participants, the STRIPES test continued indefinitely on all but one occasion. In these cases, the test was manually terminated after 25 min. We found no other descriptions in literature of participants performing the STRIPES at ceiling level or indefinite continuation of the test. A ceiling effect indicates good performance, as scoring below the lowest attainable RD indicates poor performance, but also imposes the question of whether it is possible to further expand the RD range to estimate and discriminate the scores of good and poor performers more precisely. The STRIPES has a limit of 10 RD, which translates to 2 RPO for the 5-octave stimulus used in this study (Archer-Boyd et al. 2018). O’Brien and Winn’s (2017) simulations proposed a critical cutoff point of 2.56 RPO. They argued that the current CI processors are not able to transmit the correct ripple densities above this critical limit because of the devices’ limitations in number of frequency channels and limitations in bandwidth of each channel. In this study, 10 of 15 participants scored above this critical limit of 2.56 RPO and 12 of 15 surpassed the limit of 2.0 RPO proposed for Advanced Bionics processors in a recent publication (Winn & O’Brien 2022). Preliminary simulations in our clinic also reveal at least some aliasing to be present at higher ripple densities in Advanced Bionics speech encoding strategies. However, more analyses are required to draw definite conclusions.

The claimed vowel-like cues found by O’Brien and Winn at higher RPOs on the SMRT have had no detrimental effect on the association of the SMRT with the MST when compared with the association of the MST with the STRIPES in this study. This could mean that these cues either do not significantly interfere with estimating speech recognition in noise or that the SMRT does not adequately measure spatial resolution but measures some sort of speech recognition correlate that is comparable to spatial resolution as measured on the STRIPES.

Another limitation of this study is the relatively high a priori consonant-vowel-consonant and word scores of the participants. It is possible that there would’ve been a shift in the found correlations if more poor performers had also been part of the study.

In conclusion, both the SMRT and the STRIPES possess similar test-retest reliability and revealed a similar association with the Dutch/Flemish MST. On the basis of the results of this study and the known questions regarding the validity of high RPO outcomes in CI testing, it is debatable whether spurious cues on the SMRT have had a significant effect on the association with speech recognition in noise. Both spectro-temporal ripple tests are equally capable as indicators of CI performance in relation to speech recognition in noise in studies with monopolar and steered speech-encoding strategies.

## ACKNOWLEDGMENTS

This research was supported by non-restrictive research funding from Advanced Bionics.
